# Signs of the times: universalism and localism

**DOI:** 10.1192/bji.2020.38

**Published:** 2020-08

**Authors:** John Cox

**Affiliations:** Emeritus Professor of Psychiatry, Keele University, Staffordshire, UK. Email: John6.Cox@gmail.com

**Keywords:** Perinatal mental health, person-centred care, cross-cultural psychiatry, COVID-19, international psychiatry

## Abstract

At a time when nationalism has reappeared in Europe, when COVID-19 is not yet quarantined and when compassion coexists with grief, there is a need to consider the impact of these societal changes on international collaboration, frequency and management of perinatal mental disorder – and new roles for psychiatrists and other health professionals in the digital and AI (artificial intelligence) post-COVID era.

‘On becoming pregnant a woman enters a world of uncertainties, fears and threats […] quite often anxiety wins out over veneration and contentment’, Henri Collomb (1913–1979)

These thematic papers from France,^1^ China^2^ and Africa^3^ could continue this discussion. They each provide a ray of light in a dark place and suggest care pathways for families with perinatal mental disorder that respect the past but pioneer the future. They show how to collaborate with colleagues and those with lived experience, how to improve the prospects for well-being by the integration of mental health with infant and child health services. They recommend more translational perinatal research, including economic outcomes, to underpin guidelines and to influence service managers and politicians.

## France

Sutter-Dallay and her French colleagues^1^ are rightly proud of their 19th-century clinician/researchers – Louis-Victor Marcé and Jean-Étienne Esquirol – and of their child psychiatry pioneers of mother and baby units. French contributions to psychoanalytic and ethnographic perspectives are pertinent to perinatal psychiatry.^4^

## China

In the paper from China by Schwank et al, readers are reminded of the adverse effect of the one-child policy on maternal mental health, and the strong preference for boys.^2^ The stigma of a perinatal mental disorder from within the family is prominent in Shanghai, and assessment and treatment online are the preferred care pathways.

## Africa

In Sub-Saharan Africa, Ng'oma from Malawi^3^ and other African collaborators from South Africa, Zimbabwe and Ethiopia consider the findings of an impressive range of community studies. Their paper draws attention to how the researchers working across language boundaries rode the twin tracks of the universalist etic perspective and the emic understanding of personal meaning. The etic and emic approach is derived from the linguistic terms phonetic and phonemic.^5^

## World Psychiatric Association

The WPA has urged all healthcare professionals and policy makers to improve pregnancy outcomes, reduce maternal and infant morbidity and mortality, improve the care of infants and enhance the mother–infant relationship.^6^ This relationship-based ‘body–mind–spirit’ approach to perinatal mental disorder, implicit in these three papers, can inspire us in a post-COVID-19 changing world.
